# Overlap of cognitive concepts in chronic widespread pain: An exploratory study

**DOI:** 10.1186/1471-2474-12-218

**Published:** 2011-10-05

**Authors:** Aleid de Rooij, Martijn PM Steultjens, Petra C Siemonsma, Joke A Vollebregt, Leo D Roorda, Willemine Beuving, Joost Dekker

**Affiliations:** 1 Department of Rehabilitation Research, Reade, Centre for Rehabilitation and Rheumatology (formerly Jan van Breemen Institute), Amsterdam, Netherlands; 2 School of Health, Glasgow Caledonian University, Glasgow, Scotland UK; 3 Department of Clinimetric Laboratory, Reade, Centre for Rehabilitation and Rheumatology (formerly Jan van Breemen Institute), Amsterdam, Netherlands; 4 Expertise Centre Lifestyle, TNO, Leiden, Netherlands; 5 Department of Rehabilitation, Reade, Centre for Rehabilitation and Rheumatology (formerly Jan van Breemen Institute), Amsterdam, Netherlands; 6 Department of Rehabilitation Medicine and EMGO Institute, VU University Medical Centre, Amsterdam, Netherlands; 7 Department of Psychiatry, EMGO Institute, VU University Medical Centre, Amsterdam, Netherlands

## Abstract

**Background:**

A wide variety of cognitive concepts have been shown to play an important role in chronic widespread pain (CWP). Although these concepts are generally considered to be distinct entities, some might in fact be highly overlapping. The objectives of this study were to (i) to establish inter-relationships between self-efficacy, cognitive coping styles, fear-avoidance cognitions and illness beliefs in patients with CWP and (ii) to explore the possibility of a reduction of these cognitions into a more limited number of domains.

**Methods:**

Baseline measurement data of a prospective cohort study of 138 patients with CWP were used. Factor analysis was used to study the associations between 16 different cognitive concepts.

**Results:**

Factor analysis resulted in three factors: 1) negative emotional cognitions, 2) active cognitive coping, and 3) control beliefs and expectations of chronicity.

**Conclusion:**

Negative emotional cognitions, active cognitive coping, control beliefs and expectations of chronicity seem to constitute principal domains of cognitive processes in CWP. These findings contribute to the understanding of overlap and uniqueness of cognitive concepts in chronic widespread pain.

## Background

A wide range of cognitive concepts has been proposed over the years to explain the development and persistence of chronic pain [1-3]. Relevant cognitive concepts include self-efficacy [4], cognitive coping styles [5], fear-avoidance cognitions [6,7] and illness beliefs [8,9]. These concepts are generally considered to be distinct entities, despite theoretical considerations and empirical evidence pointing towards overlap. Below, we will first shortly describe these concepts and then address the issue of overlap.

Self-efficacy is defined as one's confidence in performing a particular behaviour and overcoming barriers to that behaviour [4]. Self-efficacy is associated with activity limitations in various populations with chronic pain [10-17] and has been shown to mediate and predict beneficial outcome of rehabilitation [18,19].

Coping is defined as cognitive and behavioural efforts to manage specific external and/or internal demands that are appraised as taxing or exceeding the resources of the person [5]. Coping styles can be classified as cognitive, behavioural or physiological [20]. Examples of cognitive coping are denial of pain, positive self statements, catastrophizing [21]. Cognitive coping styles have been reported to determine patient functioning in CWP [22-24].

Fear-avoidance refers to the interpretation of pain as a signal for future pain and injury, resulting in pain-related fear and avoidance of activity. In low back pain, fear-avoidance has been argued to be more disabling than actually experienced pain [25] and is assumed to lead to activity limitation and depression [26].

Illness beliefs are ideas that patients hold about their illness [9,27] and include beliefs on the timeline, consequences and control or cure of the illness. Perceptions of serious consequences of the illness [28] and less personal control [28,29] are negatively associated with physical functioning.

Over the years relationships have been established between several cognitive concepts. For example, self-efficacy was found to be related to fear-avoidance cognitions [14]. Furthermore, an association between fear-avoidance cognitions, catastrophizing and low self-efficacy was found in subgroups of chronic pain patients [30]. In addition, in fibromyalgia patients uncertainty about the illness was found to be associated with more use of passive coping, avoidance and anxiety [31] and catastrophizing was found to be related to a limited understanding of the symptoms of FM, beliefs of a more cyclical nature of FM, and an emotional representation [32]. Moreover, in a meta-analysis relationships were shown between negative illness beliefs, emotional expressions and avoidance [28].

The cognitive concepts of self-efficacy, cognitive coping styles, fear-avoidance cognitions and illness beliefs are generally considered as theoretically distinct entities. However, given the theoretical similarities between a number of concepts (e.g. catastrophizing closely resembles emotional illness representations; and 'personal control' (illness belief) closely resemble 'perceived control of pain' (cognitive coping strategy)) and because of the empirical evidence showing associations between various cognitions, it is expected that these concepts in fact show considerable overlap.

Exploration of overlap of cognitive concepts has been identified as an ongoing challenge and exploration of this overlap is indicated [23,1]. Mikail (1993) [33], De Gagne (1995) [34] and Davidson (2008) [35] tried to understand the nature of the relationships among concepts and measurements in chronic pain. In this small body of research, a number of factors underlying chronic pain processes were found, including; affective distress, coping, support, pain and disability. In addition, Mounce (2010) [36] explored the interrelationships and overlap between negative emotional concepts relevant to chronic pain. However, little is known about the interrelationships and potential overlap between cognitive concepts derived from existing theories commonly used in chronic pain. Cognitive concepts from competing theoretical perspectives have rarely been studied simultaneously in this patient population. Although these various constructs are considered conceptually separate, they might be interrelated and there might be overlap between these concepts.

The objectives of the present study were (i) to establish inter-relationships between the cognitive concepts of self-efficacy, cognitive coping styles, fear-avoidance cognitions and illness beliefs in patients with chronic widespread pain (CWP) and (ii) to explore the possibility of a reduction of these cognitions into a more limited number of domains.

## Methods

### Patients

Data for the present study were obtained from baseline measurements of a prospective cohort study on multidisciplinary outpatient rehabilitation in CWP. Patients with CWP were referred through rheumatologists and general practitioners to the rehabilitation specialist of the pain management team of our Institute. Both a rehabilitation specialist and a psychologist assessed whether multidisciplinary treatment was indicated, following the standards of the Dutch Consensus Report of Pain Rehabilitation [37]. Inclusion criteria for the study were: age between 18 and 75 years, a diagnosis of CWP or fibromyalgia (FM) according to the International Association for the Study of Pain (IASP) [38] or the clinical criteria of the American College of Rheumatology (ACR) [39], respectively. Exclusion criteria were: pain resulting from known pathology, refusal to give informed consent and insufficient control of the Dutch language to complete questionnaires. A total of 138 patients was included in the study over a 14 month period. The ethical review board of Reade Centre for Rehabilitation and Rheumatology in Amsterdam approved this study and written informed consent was obtained from each subject.

#### Demographics and clinical variables

Demographic variables were obtained, including gender, age, pain duration, ethnicity and education. Clinical variables were obtained, including pain (Numerical Rating Scale (NRS)) [40], fatigue (subscale of the Fibromyalgia Impact Questionnaire (FIQ)) [41] and depression (Beck Depression Inventory (BDI-II)) [42].

#### Self-efficacy

The Dutch General Self-efficacy Scale (DGSS) was used to measure general self-efficacy beliefs. General self-efficacy is defined as a broad and stable sense of personal competence to deal effectively with a variety of stressful situations [43,44]. The DGSS consists of 10 items, for example 'I can always manage to solve difficult problems if I try hard enough', and 'If someone opposes me, I can find means and ways to get what I want'. Items were answered on a four point scale (1 = not at all true, 2 = hardly true, 3 = moderately true, 4 = exactly true), with a higher score indicating a higher self-efficacy. The psychometric properties of the DGSS have been documented [45,46].

#### Coping

The Dutch adaptation of the Coping Strategy Questionnaire (CSQ) was used to assess cognitive coping styles in chronic pain subjects. Seven cognitive subscales (denial of pain sensations, positive self statements, reinterpreting of pain sensations, diverting attention away from pain sensations, praying and hoping, catastrophizing, and perceived control of pain) of the CSQ were used in the present study. Per item the subject indicated to what extent this particular coping strategy was utilized. A higher score (range 0-10) indicated that the subject made more use of that particular coping strategy. The validity and reliability of the CSQ have been documented [47,48].

#### Fear-avoidance

The Tampa Scale of Kinesiophobia (TSK) was used to assess fear-avoidance cognitions i.e. self-reported fear of movement and (re)injury (e.g. 'my body is telling me I have something dangerously wrong', 'it's not safe for a person with a condition like mine to be physically active'). Items are evaluated on a 4-point Likert scale, ranging from 'strongly disagree' to 'strongly agree'. The scale consists of 17 items and a higher score indicates a higher degree of fear-avoidance. Adequate psychometric properties of this scale have been documented [49,50].

#### Illness beliefs

Seven subscales of the Revised Illness Perceptions Questionnaire (IPQ-R) were used to measure illness beliefs - chronic timeline expectancies of the illness ('timeline'); expectancies of the variability of the illness ('timeline cyclical'); expected effects and outcome of the illness ('consequences'); extent to which patients believe they can control the illness ('personal control'); belief in treatment and recommended advice ('treatment control'); patient's logical and complete understanding of the illness ('coherence'); negative emotional reactions like anger and fear related to the illness ('emotional representations'). Items in these scales are rated on a 5-point Likert scale ranging from 'strongly disagree' to 'strongly agree'. Higher scores on personal control, treatment control and low scores on illness coherence demonstrate positive beliefs about the controllability of the illness and a personal understanding of the illness, respectively. High scores on timeline, timeline cyclical, consequences and emotional representation demonstrate strongly held beliefs about the chronicity of the illness, the cyclical nature of the illness, negative consequences of the illness, and more emotional representations, respectively [51,52]. The validity and reliability of the IPQ-R have been documented [52,53].

### Statistical analysis

The distribution of scores was evaluated using descriptive statistics. Internal consistency of the (sub)scales of the measurements was evaluated (Cronbach's alpha). Cronbach's alpha above 0.70 is considered to be acceptable [54].

Correlation coefficients were computed to establish the bivariate relationships between self-efficacy, fear-avoidance, cognitive coping styles and illness beliefs. Next, an explorative orthogonal factor analysis was performed to assess whether these cognitions could be reduced to a limited set of factors. Because of a non normal distribution of subscales of the CSQ we analyzed the data of the factor analysis based on spearman correlation coefficients. As a first step, the measures for self-efficacy (DGSS), cognitive coping styles (CSQ), fear-avoidance cognitions (TSK), and illness beliefs (IPQ-R) were subjected to a principal components analysis (PCA). The number of principal components to be used in the second step was set based on the elbow of the scree plot of principal component eigenvalues. In the second step, an orthogonal varimax rotation was performed. The model resulting from this varimax rotation is presented. Only measures with a factor loadings > 0.4 were considered to load on this factor. All analyses were performed using SPSS for Windows 15.0 (SPSS Inc., Chicago, IL).

## Results

### Demographics and baseline characteristics

Of the 138 subjects included in the study, four subjects had missing values in the questionnaires and were excluded from the analyses presented below. Demographics and baseline characteristics of the study sample are shown in Table 1. Of the 134 subjects, 92.5% were female and 7.5% were male. Subjects were predominantly middle aged and of Dutch ethnicity. Patients scored high on both experienced pain intensity and fatigue. According to the BDI scores [55], 20% of the participants had minimal depression (range 0-13), 27% light depression (range 14-19), 28% moderate depression (range 20-28) and 25% serious depression (range 29-63). Cronbach's α of (sub)scales of the DGSS, CSQ, TSK and IPQ-R found in the present study, were acceptable and ranged from .72 to .92. The Cronbach's α for the subscale treatment control of the IPQ-R was found at .65.

**Table 1:** Characteristics of patients

Sex (female)	92.5%
Age, years	45.5 (10.5)
Ethnicity:	
Dutch	75.4%
Surinam/Antillean	10.4%
Other	14.1%
Education:	
Primary	17.9
Secondary	48.5%
High	33.6%
Pain duration in months	84.0 (36.0; 177.0)
NRS Pain	6.2 (2.1)
FIQ Fatigue	8.3 (1.6)
BDI-II Depression	21.3 (8.8)
IPQ Emotional representation	19.1 (4.7)
IPQ Illness coherence	15.2 (4.8)
IPQ Consequence	20.8 (4.3)
IPQ Timeline	23.5 (4.0)
IPQ Timeline cyclical	15.2 (3.2)
IPQ Personal control	18.6 (4.4)
IPQ Treatment control	16.2 (2.9)
CSQ Perceived pain control	4.9 (2.4)
CSQ Catastrophizing	6.1 (2.8)
CSQ Praying and hoping	4.0 (2.0; 6.0)
CSQ Positive self statements	5.0 (2.0; 7.0)
CSQ Denial pain sensations	6.0 (2.0; 8.0)
CSQ Diverting attention	4.0 (2.0; 8.0)
CSQ Reinterpreting pain	3.0 (1.0; 7.0)
TSK Fear-avoidance	28.4 (7.0)
DGSS General self-efficacy	2.90 (0.6)

Values are means (SD), medians (IQR) or percentages, BDI = Beck Depression Inventory, CSQ = Coping Scale Questionnaire, DGSS = Dutch, General Self-efficacy Scale, FIQ = Fibromyalgia, Impact Questionnaire, IPQ = Illness Perception Questionnaire, NRS = Numerical Rating Scale, TSK = Tampa scale for Kinesiofobia

### Bivariate correlations between self-efficacy, coping cognitions, fear avoidance and illness beliefs

Spearman correlation coefficients were low to moderate, ranging from .01 to .64 (see Table 2). Correlations higher than > .34 will be described below. General self-efficacy was related to the cognitive coping style 'positive self statements'(i.e. .42) and the illness beliefs 'emotional representation' (i.e. -.45) and 'personal control' (i.e. .35). In addition, the cognitive coping style 'catastrophizing' was related to the illness belief 'emotional representation'(i.e. .37). Furthermore, the cognitive coping style 'perceived control' was found to be related to the illness belief 'personal control' (i.e. .39). Fear-avoidance cognitions were related to the cognitive coping style 'praying and hoping'(i.e. .34) and the two illness beliefs 'consequences' (i.e. .34) and 'emotional representations' (i.e .34).

**Table 2:** Spearman correlation coefficients among general self-efficacy beliefs, cognitive coping strategies, fear-avoidance cognitions and illness beliefs


**measures**^**a**^	**1** **Gen.** ** self-** **efficacy**	**2** **Catastro-** **phizing**	**3** **Perceived ** **control**	**4** **Denial ** **of** **pain**	**5** **Pos.** **self ** **statements**	**6** **Reinter- preting** **pain**	**7** **Pray.** **hoping**	**8** **Div.** **atten-** **tion**	**9** **Fear-** **avoid-** **ance**	**10** **Conse-quence**	**11** **Pers.** **control**	**12** **Coher-** **ence**	**13** **Emo.** **repr**	**14** **Timel**	**15** **Timel.** **cycl**	**16** **Treatm.** **control**

**1**																
**2**	-.23(**)															
**3**	.11	-.03														
**4**	.22(*)	-.06	.28(**)													
**5**	.42(**)	-.11	.18(*)	.64(**)												
**6**	.05	.11	.33(**)	.39(**)	.28(**)											
**7**	-.17(*)	.45(**)	.07	.12	.08	.37(**)										
**8**	.09	.13	.32(**)	.41(**)	.50(**)	.49(**)	.31(**)									
**9**	-.16	.33(**)	-.01	-.12	-.14	.02	.34(**)	-.09								
**10**	-.21(*)	.19(*)	-.10	-.18(*)	-.11	.12	.22(*)	.05	.34(**)							
**11**	.35(**)	-.10	.40(**)	.32(**)	.27(**)	.04	-.15	.15	-.14	-22(**)						
**12**	-.29(**)	.28(**)	-.24(**)	-.10	-.19(*)	.06	.20(*)	-.05	.24(**)	.30(**)	-.23(**)					
**13**	-.42(**)	.37(**)	-.05	-.26(**)	-.22(*)	.16	.31(**)	.03	.34(**)	.56(**)	-.17	.53(**)				
**14**	-.08	.22(**)	-.10	-.05	-.00	-.03	.01	-.02	.08	.31(**)	-.24(**)	.04	.11			
**15**	.13	.03	-.12	.05	-.00	.06	-.01	-.03	-.02	.13	.06	.20(*)	.15	.17		
**16**	.06	-.04	.16	.19(*)	.22(*)	.03	.03	.12	-.09	-.13	.42(**)	-.14	-.08	-.19(*)	.08	

Note. * p < .05, ** p < .01, ^a ^1 = (DGSS) general self efficacy, 2 = (CSQ) catastrophizing, 3 = (CSQ) perceived control of pain, 4 = (CSQ) denial of pain sensations, 5 = (CSQ) positive self statements, 6 = (CSQ) reinterpreting of pain sensations, 7 = (CSQ) praying and hoping, 8 = (CSQ) diverting attention away from pain sensations, 9 = (TSK) fear-avoidance, 10 = (IPQ) consequence, 11 = (IPQ) personal control, 12 = (IPQ) coherence, 13 = (IPQ) emotional representation, 14 = (IPQ) timeline, 15 = (IPQ) timeline cyclical, 16 = (IPQ) treatment control

### Factor analysis of self-efficacy, cognitive coping styles, fear avoidance cognition and illness beliefs

Based on the factor analysis, three factors were identified, which explained 48% of the total variance. The eigenvalues of the three factors were 3.5, 2.7 and 1.4, respectively. The first factor included three illness beliefs (IPQ-R): 'illness coherence', 'consequence of the illness' and 'emotional representation'; the cognitive coping styles (CSQ) 'catastrophizing' and 'praying and hoping'; fear-avoidance cognitions (TSK); and general self-efficacy beliefs (DGSS) (see table 3). The negative loading of self-efficacy (i.e. less self-efficacy) is in line with positive loadings of the other items (e.g. more catastrophizing). The second factor was made up of cognitive coping styles (CSQ): 'perceived control over pain', 'denial of pain sensations', 'positive self statements', 'reinterpreting of pain sensations' and 'diverting attention away from pain sensations'. The third factor was made up of the illness beliefs (IPQ-R): 'personal control' and 'treatment control'; the cognitive coping style (CSQ) 'perceived control over pain'; and the illness beliefs (IPQ-R) 'consequence of the illness', 'timeline' and 'timeline cyclical'. The negative loadings of the control items (i.e. less control) are in line with the positive loadings of the other items (e.g. expectations of chronicity).

**Table 3:** Results of factor analysis

Items	Factor		
	1	2	3
IPQ Emotional representation	.77		
IPQ Illness coherence	.55		
IPQ Consequence	.55		.43
IPQ Timeline			.67
IPQ Timeline cyclical			.54
IPQ Personal control			-.43
IPQ Treatment control			-.40
			
CSQ Perceived pain control		.45	-.49
CSQ Catastrophizing	.63		
CSQ Praying and hoping	.65		
CSQ Positive self statements		.77	
CSQ Denial pain sensations		.76	
CSQ Diverting attention		.75	
CSQ Reinterpreting pain		.67	
			
TSK Fear-avoidance	.59		
DGSS General self-efficacy	-.58		

Eigenvalue	3.5	2.7	1.4

Explained variance	22.2%	16.9%	8.5%

Model accounted for 47.6% of the total variance. Only factor loads > .40 are listed.

A series of sensitivity analyses was performed. First of all, the same factor analysis as above was performed using oblique rotation. This resulted in a scree plot and factor solution which were similar to the analysis based on the orthogonal factors described above. The correlations between the oblique factors were low (ranging from .07 to -.12). Secondly, a series of sensitivity analyses (using varimax rotation) was performed with a reduced number of variables. Each time, the presence of variables related to one specific cognitive theory was reduced (e.g. an analysis was done without the TSK to check the factor structure of the remaining cognitions in the absence of a fear-avoidance measure). This resulted in the same factor structure each time. Finally, an analysis was done with a reduced number of variables of the CSQ to reduce the impact of shared method variance on the second factor found in the original analysis. Again the factor structure presented in this paper was maintained.

## Discussion

To our knowledge this is the first study to purposefully explore overlap between cognitive concepts in CWP. Sixteen concepts derived from the subscales of 4 measurements, all having good psychometric properties, were submitted to an exploratory factor analysis. The cognitive concepts were derived from various theoretical models and are commonly used in reference to chronic pain. Our measurements were conducted using a representative population of chronic widespread pain and FM patients, characterized by pain, fatigue and depression. The present results suggest that a limited number of underlying domains can be distinguished among measurements of the cognitive concepts of self-efficacy, cognitive coping styles, fear-avoidance and illness beliefs.

The first factor can be interpreted as the domain of *negative emotional cognitions*: illness coherence, consequences of the illness, emotional representation, catastrophizing, praying and hoping, fear-avoidance cognitions and low general self-efficacy. The second domain concerns *active cognitive coping*: denial of pain sensations, perceived control over pain, positive self statements, reinterpreting of pain sensations and diverting attention away; these concepts are all referring to the use of active coping cognitions. The third domain consists of cognitions of *control beliefs and expectancies of chronicity of the illness*: personal control and treatment control, perceived control over pain, consequences of the illness and expectations of chronicity and beliefs regarding variability of the illness. Sensitivity analyses showed these three domains to be stable when a different approach to factor analysis was chosen or the number of cognitions entered into the analysis was changed. Thus, our data suggest three domains of cognitive concepts in CWP.

The findings with regard to the first factor suggest that there is overlap between the concepts of fear-avoidance cognitions, catastrophizing low self-efficacy and negative illness beliefs. This finding is consistent with previous studies reporting associations between self-efficacy, fear-avoidance and catastrophizing [14,30], uncertainty about the illness and the use of passive coping, avoidance [31], catastrophizing and a limited understanding of the symptoms of FM and emotional representation [32], negative illness beliefs, emotional expressions and avoidance [28]. However, present results go one step further and suggest that all these cognitions should be regarded as entities within the domain of 'negative emotional cognitions'.

We found that low general self-efficacy is related to negative emotional cognitions (first factor) and not to specific illness-related control beliefs (third factor). Possibly, a distinction should be made between general self-efficacy (defined as a broad and stable sense of personal control) and specific illness-related control beliefs and self-efficacy beliefs (defined as the confidence in being able to perform a particular behaviour or task despite of pain). General self-efficacy (assessed with items such as 'I can always manage to solve difficult problems if I try hard enough') and more specifically -the lack of general self-efficacy seem to belong to the domain of negative emotional cognitions.

The cognitive coping style 'praying and hoping' was also part of the first factor which otherwise mainly comprised negative emotional cognitions. However, praying and hoping does not directly reflect a negative and emotion-based view of chronic illness. If replicated, further research is required to explain the association between praying and hoping on the one hand and negative emotional cognitions on the other.

The findings with regard to the second factor show that active coping cognitions, can be distinguished from negative emotional cognitions on the one hand and control beliefs and expectations on the chronicity of the illness on the other hand. This finding supports the findings of other studies [47,56,57]. Our results confirm that active coping styles can be distinguished from other cognitive concepts. It seems that active coping styles constitute the second principal domain of cognitive processes in the assessment of CWP.

It should be acknowledged that the second factor consists of subscales from one coping questionnaire. The similarity of the format of the subscales may have contributed to the observed associations (method variance). This criticism does not apply to the first and the third factor, as subscales from various questionnaires loaded on these factors. Future research should aim to circumvent shared method variance as an explanation for the association of subscales.

The finding with regard to the third factor suggest that in CWP expectations of personal control over the illness and treatment control (i.e. belief in the successful management of the illness by treatment) are closely related to each other and belong to the same domain. Moss-Morris (2002) [52] have commented that in illnesses with a variety of treatment options and no established curative pathways, treatment control may closely resemble personal control due to the level of personal choice in the treatment process. This might explain the close relationship between personal and treatment control beliefs found in the CWP population of the present study. In addition, it is comprehensible that control beliefs on the one hand and expectations of chronicity on the other hand load inversely on the same factor in our analyses: patients who believe to have control may expect a relatively fast recovery. The results suggest that control beliefs and expectations of chronicity constitute the third principal domain of cognitive processes in the assessment of CWP.

Davidson (2008) [35] used a similar approach, i.e. factor analysis, to analyse the association between concepts in "the chronic pain experience". The findings of Davidson, show some similarity to our results: negative emotional cognitions and coping cognitions were also identified as domains within the pain experience. It should be noted however that in their study the association between a wide range of concepts (e.g. disability, fear-avoidance, catastrophizing, cognitive and behavioural coping, psychological functioning, depression) in chronic pain was assessed. Our study focussed on a much more limited range of concepts; we focussed on the association between cognitive concepts only.

The present study has some limitations. Firstly, a concern of the present study may be the size of the sample. However, as both the factor analysis and sensitivity analysis resulted in a well interpretable, robust facture structure it is unlikely that the sample size has seriously distorted the results. Secondly, internal validity of the scales used was acceptable. Only the internal validity of the subscale treatment control of the IPQ-R was just below the recommended cut off: this may have led to a higher measurement error and an underestimation of the true relationships. Thirdly, we used a general self-efficacy scale to measure self-efficacy expectations in a chronic pain population. Self-efficacy beliefs can be distinguished in general self-efficacy and specific self-efficacy beliefs (e.g. pain self-efficacy). General self-efficacy refers to a broad stable sense of personal competence to deal effectively with a variety of stressful situations [43,44]. Pain self-efficacy beliefs refers to confidence in performing activities while in pain [58]. General self-efficacy expectations are less commonly used in the measurements in chronic pain than specific pain self-efficacy expectations. It seems recommendable that in future research both measurements are integrated into the model to see to which domain they belong.

In our opinion, the option of reduction of a number of cognitive processes and a more parsimonious theory on cognitive processes in chronic pain has thus far not been pursued on a sufficiently serious basis. More understanding of the extent of overlap and uniqueness of cognitive concepts and a more integrative theory is worthwhile for patients, clinicians and researchers. Our finding of three dimensions offers a good starting point. The results indicate that it will be meaningful to investigate on a wider scale whether negative emotional cognitions, active coping styles, control beliefs and expectations of chronicity constitute principal domains of cognitive concepts in CWP. However, in view of the diversity (e.g. not all cognitive concepts were included in our analysis) and complexity of cognitive processes in CWP and the explorative nature of our analysis, it is as yet too early to reconstruct and reformulate a new and integrative model of cognitive processes in CWP. Replication, confirmatory analyses and longitudinal studies will be needed to further validate the grouping of different cognitions into a limited set of domains.

## Conclusions

The results of this study indicate that negative emotional cognitions, active cognitive coping, control beliefs and expectations of chronicity seem to constitute principal domains of cognitive processes in CWP. These findings contribute to the understanding of overlap and uniqueness of cognitive concepts in chronic pain.

## Competing interests

The authors declare that they have no competing interests.

## Authors' contributions

JD, MS and AR designed the study. AR coordinated the data collection, performed the statistical analysis. AR, JD and MS wrote the manuscript. PS collected data, and helped to draft the manuscript. JV, WB, LR participated in the design of the study and helped to draft the manuscript. All authors read and approved the final manuscript.

## Pre-publication history

The pre-publication history for this paper can be accessed here:

http://www.biomedcentral.com/1471-2474/12/218/prepub
